# Evaluation of the healthy median nerve elasticity: Feasibility and reliability of shear wave elastography

**DOI:** 10.1097/MD.0000000000012956

**Published:** 2018-10-26

**Authors:** Bihui Zhu, Feng Yan, Ying He, Liyun Wang, Xi Xiang, Yuanjiao Tang, Yujia Yang, Li Qiu

**Affiliations:** a Department of Ultrasound; b Clinical Ultrasound Imaging Drug Research Lab, West China Hospital of Sichuan University, Chengdu, Sichuan Province, China.

**Keywords:** elasticity, inter- and intraobserver agreements, median nerve, shear wave elastography

## Abstract

The present study applied the shear wave elastography (SWE) to the median nerve in order to investigate the feasibility and reliability of its use in 40 healthy volunteers. Shear wave velocities of the median nerve on bilateral forearms and right carpal tunnel were obtained with relaxing or stretching conditions. The inter- and intraobserver agreements and differences of nerve elasticity among groups were evaluated using intraclass correlation coefficients, the paired *t* test, and the Wilcoxon signed-rank test, respectively. The stiffness of the site was expressed by 3 types of values: mean, minimum, and maximum shear-wave velocities. The inter- and intraobserver agreements were excellent (0.852–0.930) on the right forearm. No differences were detected between the bilateral forearm (mean: *P* = .14), while the values of different body sites and postures were statistically different (*P* < .001). SWE, as a noninvasive and objective tool, reached a good consistency in evaluating the healthy median nerve. Further studies are essential to investigate the detailed influencing factors and provide an insight of SWE to estimate both the normal nerve and peripheral neuropathy.

## 1 Introduction

In the peripheral nervous system, the connective tissue is constituted of 3 layers: the epineurium, the perineurium, and the endoneurium. The axon, basic component of peripheral nerves, is wrapped by the endoneurium. Groups of these axons form the nerve fascicles surrounded by the perineurium that contains collagen fibers, fibroblasts, and vessels, which in turn are joined together within another layer known as the epineurium. The anatomy endows peripheral nerves with viscoelasticity, and this mechanical property may protect the nerve to resist mechanical stress, as well as provide abundant information about diagnosis and classification of peripheral nerve pathologies.^[1–3]^ Peripheral neuropathies refer to a group of disorders and can be divided broadly into traumatic and nontraumatic neuropathies, including entrapment, inflammation, neoplasm,^[4]^ and complication of systemic diseases.^[5–7]^

Previous studies have investigated the value of existing diagnostic methods of peripheral nerve pathologies. The diagnosis is initially on the basis of characteristic symptoms, but an asymptomatic presentation can occur in patients.^[6,7]^ Electrophysiological test is considered the reliable method for peripheral neuropathies, but just efficiently for the abnormalities of large nerve fibers, and cannot detect small fiber neuropathy because the absence or reduced myelin of small fibers results in slow conduction velocities that are beyond the resolution of these studies.^[8–10]^ In addition, this assay is as time-consuming and expensive as magnetic resonance imaging.^[11–14]^ Since the first report of the application of ultrasonography (US) for carpal tunnel syndrome in 1992, US examinations have been used as a complementary test for peripheral nerve pathologies.^[1]^ The peripheral nerve has a relatively mixed hyperechoic and hypoechoic appearance in US, which provides a noninvasive assessment of peripheral nerve pathologies with respect to morphology and localization.^[15,16]^ Although there are advantages of the nerve US, the qualitative result and wide range sensitivity and specificity limit the potential development.^[14]^

Thus, utilizing the elastography ultrasound (EUS) to quantitatively assess the elasticity of the peripheral nerve may serve as an alternative method. The structure and composition of the tissue determine its deformation and rehabilitation capability, which is characterized by the elasticity. EUS is a technique based on this biomechanical property that evaluates the elasticity of the tissues after an external or internal stimulus imposed on the target tissues.^[16]^ Currently, several elastography techniques are applied, such as strain EUS, shear wave EUS (SWE), acoustic radiation force impulse EUS (ARFI), and transient EUS.^[17]^ Although strain EUS is the most common and cost-effective technique, the result is qualitative and largely dependent on the examiners.^[18]^ ARFI is an alternative type of strain EUS that does not use any mechanical pressure. It generates a shear wave in the target area by focused ultrasound beam and acquires a quantitative result with shear wave velocities. SWE is equipped with both color-coded qualitative elastograms and quantitative maps of shear wave velocity.^[19]^ This method is based on the different physical principles stating that the shear wave is caused by the ultrasound impulse. In addition, the shear wave velocity, such as ARFI, is calculated automatically in a region of interest (ROI) in this map for the tissue stiffness. Simultaneously, the Young module of elasticity is obtained by the formula *E* = 3∗*V*^2^, where *E* is Young module in kPa and *V* is the shear wave velocity in m/s.

Although several studies describe EUS for the evaluation of the peripheral nerves in peripheral neuropathies, such as carpal tunnel syndrome or diabetic peripheral neuropathy, SWE is considered more objective than strain EUS and still less utilized.^[20]^ The present study used SWE to explore the reliability and feasibility of measuring the median nerve elasticity in healthy volunteers. In order to gain a better understanding of SWE in the detection of nerve elasticity and provide the methodological basis for the further study of peripheral nerves in pathological conditions, the intra- and interobserver reproducibility, the difference in nerve stiffness of different postures, the difference between right wrist and right forearm, and the difference in the bilateral forearms were investigated.

## 2 Materials and methods

### 2.1 Participants

A total of 40 healthy subjects were enrolled in the present study from November 2016 to April 2017. Volunteers were excluded if they presented a history of systemic neurological disorders, post-traumatic changes to nerves, nerve tumors, nerve entrapment syndromes, musculoskeletal disorders, or other systemic metabolic disease. The study was approved by the West China Hospital of Sichuan University Ethics Committee, and informed consent was obtained from all participants.

### 2.2 Positioning

**Figure 1:**
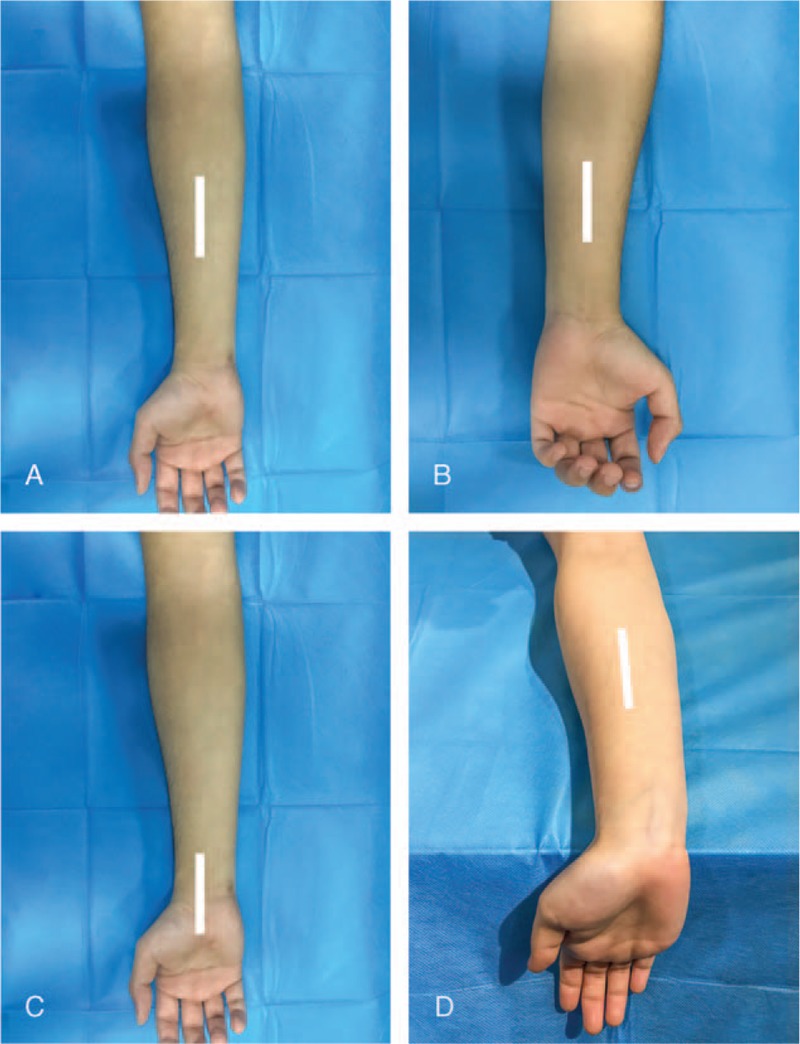
Examples of median nerve SWE measurements on different locations and postures. (A, D) Right mid-forearm. (B) Left mid-forearm. (C) Right wrist. (A–C) Relaxing condition, posture 1. (D) Stretching condition, posture 2. White lines are the locations of the probe.

During examinations, the room was temperature-controlled and silence maintained. For imaging of the median nerve, each subject was asked to sit facing the examiner with the arm extended. Initially, the elbow was flexed 90°, the forearm was in the supine position, and wrists relaxed on a flat surface with fingers semi-flexed (posture 1; Fig. 1A). Then, the subject was instructed to another posture, wherein the wrist was stretched maximally while maintaining the forearm on the flat surface (posture 2; Fig. 1D). This position was chosen owing to the lengthening of the median nerve. The examined limb was carefully maintained in a neutral position without any movement.

### 2.3 Equipment and SWE measurement

**Figure 2:**
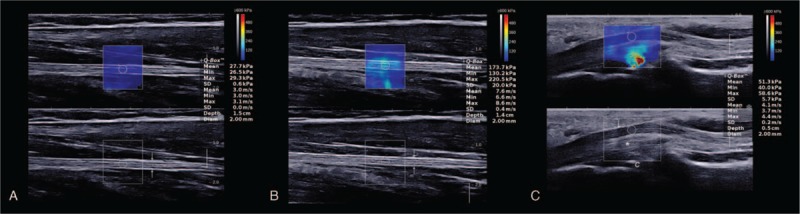
Shear wave elastography images of the median nerve from a 24-year-old healthy female. (A, B) Longitudinal images of the median nerve (between arrows) on the right mid-forearm corresponding to postures 1 (A) and 2 (B). Note the increase in stiffness value from relaxing (mean, 3.0 m/s) to stretching condition (mean, 7.6 m/s). (C) SWE image of the median nerve (white arrows) obtained at the proximal inlet of the carpal tunnel on the right side. The mean elasticity is measured as 4.1 m/s. According to the color box, blue color represents the minimal stiffness (0 m/s) and red color represents the maximal stiffness (16.3 m/s). *C* proximal carpal row, ^∗^ flexor tendons.

All elastography examinations were performed by Aixplorer ultrasound scanner (SuperSonic Imaging, Aixen-Provence, France) with a 4 to 15-MHz linear array probe. Light contact was applied to the transducer onto the skin surface using the coupling agents, avoiding a compression effect. The transverse imaging plane of the nerve with B-mode was identified initially. Then, the transducer was rotated 90° to obtain the longitudinal imaging plane, which is a parallel orientation to the nerve, followed by the SWE mode. During preliminary studies, we found that reliable elasticity data could not be acquired in transverse orientation. A superficial musculoskeletal setting was chosen during the elastographic evaluation. Considering the anisotropy of nerve, shear wave velocities were determined for the nerve stiffness that does not alter with different modes and scale in the case of a completely filled square ROI as described previously.^[17]^ And the range of velocities that can be measured by this US system is 0 to 16.3 m/s. For the best image quality, the scale was adjusted to 600 kPa with “standard” or “penetration” mode, the size of ROI was kept as 2 mm, and the depth was fixed at 2 cm. Figure 2 represents the typical SWE images.

In this study, the median nerve was assessed over the bilateral mid-forearm and proximal inlet of the carpal tunnel on the right side. Two sonographers, trained in SWE, conducted the full examinations, respectively. The median nerve images were obtained by sonographer A, and sonographer B measured the median nerve on the right mid-forearm for interobserver repeatability. Within 1 week after the first examination, the same subjects were rechecked by sonographer A, and the data were used to calculate the intraobserver repeatability. Twenty healthy volunteers were randomly chosen to be examined for the inter- and intraobserver repeatability. The 2 sonographers were blinded to the results assessed by another expert. All measurements were carried out 3 times and the average of mean, minimum, maximum velocities (m/s) data (which is *V*mean, *V*min, *V*max) were analyzed for statistical significance.

### 2.4 Statistical analysis

Statistical analyses were performed using SPSS software (Version 20.0; IBM, NY). Data were expressed as mean ± standard deviation. The Shapiro–Wilk test was used for normal distribution data. The differences in age and body mass index in volunteers were evaluated using the Mann–Whitney *U* test. A paired *t* test was used for evaluating the difference in the nerve stiffness of bilateral forearm. The median nerve shear-wave velocities of right forearm in different positions, as well as, the difference in nerve stiffness of right wrist and forearm were compared using the Wilcoxon signed-rank test, the intra- and interobserver agreements were assessed with single-measure intraclass correlation coefficients (ICCs) calculated using a 2-way random effects model. An ICC > 0.75 was indicative of excellent agreement.^[21]^ Pearson correlation was used in the correlation analysis. *P* < .05 was considered as the statistically significant difference.

## 3 Results

### 3.1 Demographic data

Table 1 summarizes the baseline demographic data of the study participants. The mean age of the participants was 31.20 ± 8.92 (range, 20–52) years and the mean body mass index was 21.72 ± 2.67 (range, 16.8–28.37) kg/m^2^. Statistical analysis did not reveal any significant difference in age, although males had a significantly higher body mass index than females.

**Table 1:**
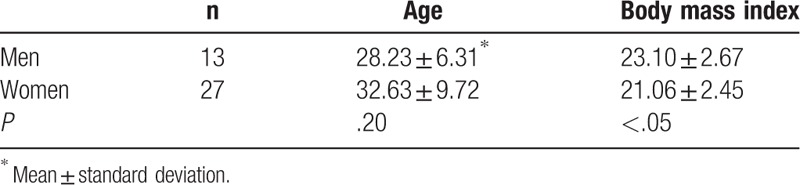
Baseline characteristics of the healthy volunteers enrolled in this study.

### 3.2 Inter- and intraobserver repeatability of median nerve measurements

**Figure 3:**
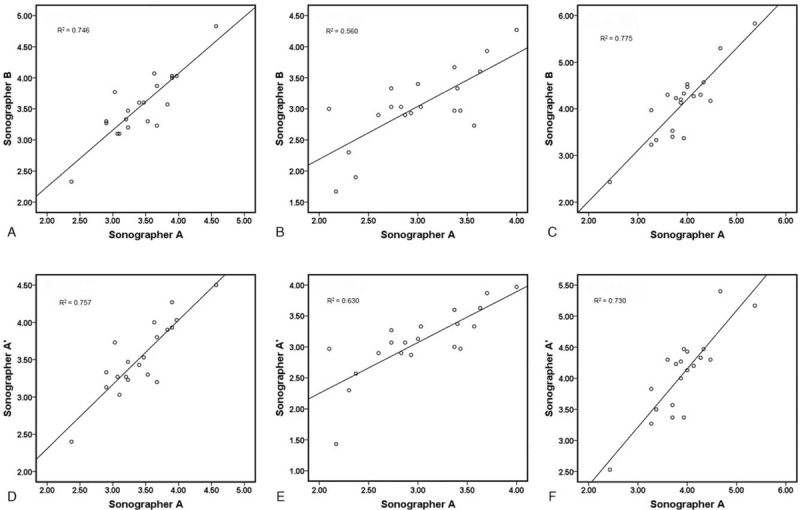
Inter- and intraobserver reliability of shear wave elastography. (A–C) The interobserver reliability of mean, minimum, and maximum elasticity values in the right mid-forearm. (D–F) The intraobserver reliability of mean, minimum, and maximum elasticity values in the right mid-forearm.

The results of inter- and intraobserver repeatability of SWE measurement in 20 volunteers are presented in Tables 2 and 3, respectively. Figure 3 shows the correlation of ICCs. As ICCs analyzed for the median nerve in the right forearm ranged from 0.852 to 0.930, an excellent inter- and intraobserver agreement was noted. *R*^2^ ranged from 0.560 to 0.775, indicating a good correlation. All the *P* values were <.001.

**Table 2:**
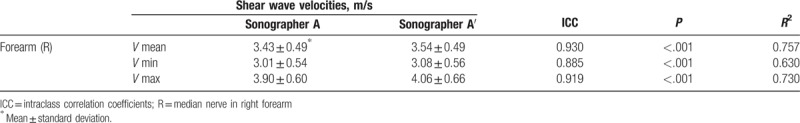
ICCs for intraobserver reliability of shear wave velocities obtained from the median nerve.

**Table 3:**
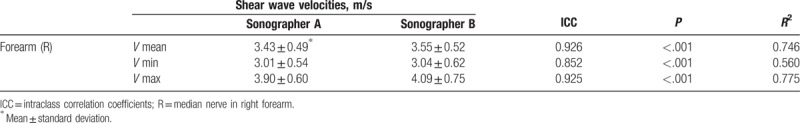
ICCs for interobserver reliability of shear wave velocities obtained from the median nerve.

### 3.3 Difference in elasticity values of median nerve on bilateral forearms

With respect to the median nerve stiffness in the same individual, the difference of nerve shear wave velocities (*V*mean, *V*min, *V*max) between bilateral forearms was calculated as *D* values; no statistical significance was observed (*P* > .05) (Table 4).

**Table 4:**
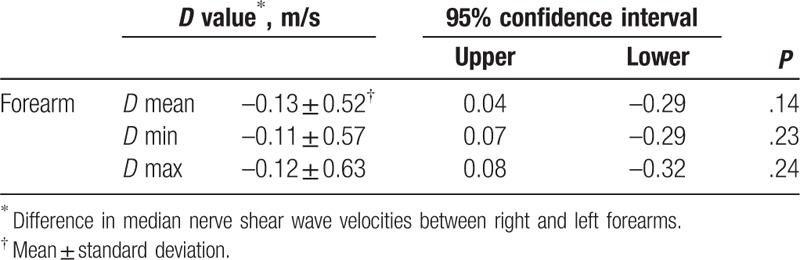
*D* values of median nerve elasticity in bilateral forearm.

### 3.4 Difference in the same median nerve stiffness of different body sites

Average shear wave velocities of the same median nerve in the different body sites are summarized in Table 5. The median nerve of the right wrist was significantly stiffer than that of the right forearm (*P* < .001).

**Table 5:**
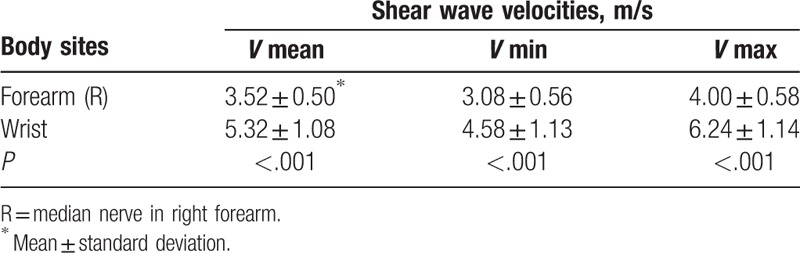
Elasticity values of the same median nerve in different body sites.

### 3.5 Difference in right forearm postures on median nerve stiffness

**Figure 4:**
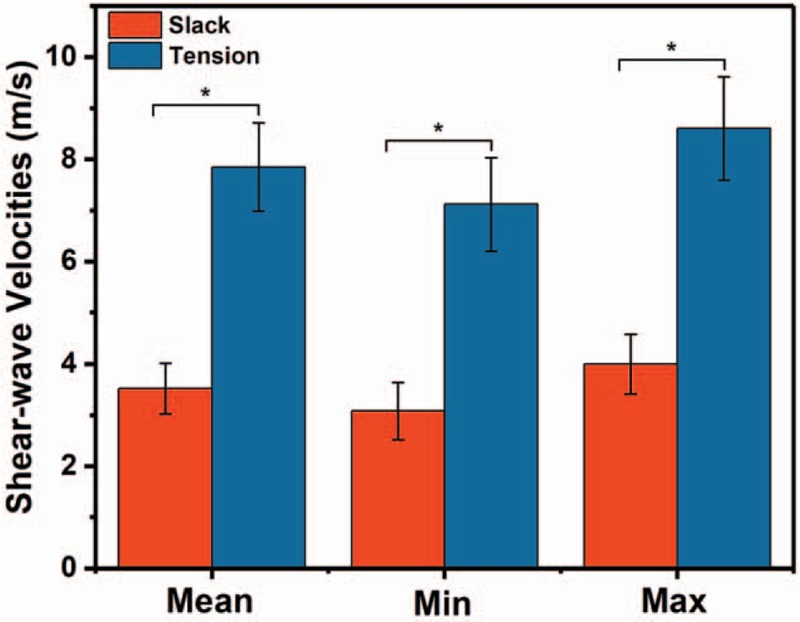
Mean, minimum, and maximum shear-wave velocities in the right mid-forearm with relaxing and tension conditions. ^∗^*P* < .001 for 3 independent elasticity values between different postures.

Shear wave velocities for the median nerve in the right forearm were obtained with 2 postures. A significant effect of the different nerve postures was observed, and the median nerve in the tension condition had a higher stiffness than that in the slack condition (*P* < .001). The data are illustrated in Fig. 4.

## 4 Discussion

The SWE has been extensively employed to assess the liver, breast mass, and musculoskeletal tissues,^[18,20,22,23]^ and increasingly used in the peripheral nerves in recent years.^[14,24]^ However, the feasibility and reliability of using SWE for peripheral nerves have not yet been investigated. In this study, we evaluated the shear wave velocities as the elasticity of the normal median nerves and focused on the feasibility and reliability for the right forearm median nerve. The measurement with SWE obtained excellent ICCs (all ICC values > 0.8) for 3 independent values—mean, minimum, maximum elasticity,^[25]^ thereby indicating good intra- and interobserver reliability, which demonstrated that this measurement was relatively little dependent on operators. The results were in agreement with the previous studies that showed the near perfect ICCs in imaging phantom.^[26]^ Recent studies applied SWE for assessing the muscle elasticity^[27–29]^ and showed satisfactory ICCs. Similar to the structure of the muscle and tendon with different fiber orientation, this technique implied that it might also provide reliable measurements for median nerve as the anisotropic tissue. Kantarci et al^[14]^ demonstrated that the interobserver variability of SWE in the healthy median nerve was excellent with an ICC of 0.848, which was in agreement with the current results. Thus, we speculated that the stable elasticity of peripheral nerves could be achieved by SWE in this standardized method.

The anatomy of peripheral nerve formation and distribution can vary, and the median nerve is one of the nerves that shows multiple variabilities. Knowledge of such variations between bilateral nerve is important for evaluation of peripheral neuropathies without misinterpretation of clinical symptoms.^[30]^ In our study, the shear wave velocities of the median nerve in symmetric left and right forearms on healthy volunteers were compared with confirm the validity of using the intrasubject contralateral normal site as the control. We found no statistically significant differences between the bilateral forearm median nerves as summarized in Table 4; this result was similar to that of Andreisek et al.^[31]^ Moreover, these results were expected as the bilateral nerves have similar anatomic structure; thus, the stiffness should be similar, and the difference in the stiffness may arise from the lesions.^[32]^ Therefore, the present finding renders that contralateral nerve elasticity might be used as the internal control for the assessment and treatment of the disease. Although in some conditions, the contralateral nerve can be easily affected, the range of normal values gained from healthy volunteers may help in defining the abnormal nerve elasticities in the further study.

Previous literatures seldom investigated the same median nerve stiffness of different body sites. In this study, the median nerve on the right carpal tunnel and mid-forearm were compared; the shear wave velocities were found to differ significantly between the 2 body sites (*P* < .001); the nerve stiffness of the wrist was higher. In contrast to the forearm, the structure of the carpal tunnel is believed to be complicated, and nerve stiffness may be easily influenced by surroundings where the nerve approaches the bone surface,^[33]^ which could be one of the factors contributing to the present phenomenon. In addition, the median nerve depths on the 2 sites were different. Although the probe pressure was similar, the superficial probe might exert excess pressure, partially explaining the difference. Also, the nerve elasticity value on the carpal tunnel was dependent on the measurement location,^[34]^ which gave it more variability than that on the forearm. This finding suggests that the nerve stiffness of different body sites may be different and selecting the suitable site for SWE-based measurement of peripheral nerve may provide reliable results.

The nerve elasticity demonstrated an increase in stretching conditions in this study. Under tension condition, the mean shear wave velocity for the right forearm was 7.85 ± 0.87 m/s, which is 2-fold that of the relaxing posture. Greening and Dilley^[35]^ talked about the movement-evoked changes of the nerve shear wave velocities, and the conclusion confirmed our result. However, they just mentioned the mean value, we got the minimum and maximum elasticity values showing the same increase, which more comprehensively proved the influence of the limb posture. It was also consistent with the literature,^[36]^ wherein the stiffness of resting and contracting muscle was attributed to the phenotype of the anisotropic tissue. Thus, the significant variation in median nerve elasticity in different limb positions suggests that caution should be exerted with limb postures during examination for precise outcomes. In addition, this study compared bilateral median nerve before and no significant difference was observed. This would help patients with restricted limb posture to be examined using contralateral side, which other literatures seldom considered.

Nevertheless, our study has several limitations. First, the study population is small and consists of only healthy volunteers. Although comparing the disease with normal condition is crucial, it mainly explores the methodological basis of peripheral nerves for further studies. In addition, with respect to the peripheral neuropathy, not only peripheral nerves of upper arms but also of lower limbs are affected. In the current study, we performed measurements on the median nerve, and hence, it is essential to discuss the tibial nerve elasticity assessment in depth.

## 5 Conclusion

The present study evaluated the feasibility and reliability of the median nerve elasticity with SWE and acquired high reproducibility and reliability. Moreover, the obvious changes were detected for different body sites and postures. However, no significant difference was observed between bilateral median nerves and contralateral nerve, which might be used as the internal control. These results could provide a methodological basis for further studies, which are essential to investigate the detailed influencing factors of nerve stiffness and evaluate both the normal nerve and peripheral neuropathy.

## Author contributions

**Methodology:** Bihui Zhu, Liyun Wang, Li Qiu.

**Project administration:** Li Qiu.

**Resources:** Ying He, Li Qiu.

**Writing – original draft:** Bihui Zhu.

**Writing – review & editing:** Feng Yan, Xi Xiang, Yuanjiao Tang, Yujia Yang, Li Qiu.
